# Design, implementation and first results of a 3RD generation digital photogrammetric system from trunk surface assessment and scoliosis screening

**DOI:** 10.1186/1748-7161-7-S1-P14

**Published:** 2012-01-27

**Authors:** TB Grivas, P Patias, K Soultanis, E Stylianidis, V Tsioukas, C Georgiadis, C Andreou, P Charalambous, Y Chrysanthou

**Affiliations:** 1 “Tzanio” General Hospital of Piraeus, Pirae Brilissia, Greece; 2 “Tzanio” General Hospital of Piraeus, Pirae Brilissia, Greece

## Background

Scoliosis patients typically undergo numerous spinal radiographs and exposed to relatively high doses of ionizing radiation. This has raised concern regarding the effects of this repeated exposure. Additionally assessment of spinal deformities using surface topography of the back is currently considered essential. Digital Photogrammetry can contribute in non-invasive measurements of the patient’s back and 3D reconstruction of surface shape from digital photos.

## Materials and methods

The design, technical approaches, involved technologies, software development and instrumentation of an innovative portable system is described. The system is based on a high-resolution digital camera, a range camera and a portable computer. The measurement protocol is highly automated, in order to minimize error sources and maximize user friendliness.

## Results

The implementation and the first evaluation results of the developed system are presented. The 3D reconstruction of the back’s surface is realized with high accuracy. The system can document the results of conservative or surgical treatment for spinal deformities and will be useful for screening purposes. Spinal deformations indices are derived and clinically tested for evaluation against the Cobb angle radiographic measurements, which are considered the “golden standard” in scoliosis assessment.

## Conclusions

The quest to elimination of radiation exposure of scoliotic patients gave rise to a number of non-invasive diagnostic techniques. Based on recent technological advances and on a better understanding of medical needs, the system presented, automated to a high degree, produces medically meaningful indices for pre- and post-treatment posterior trunk surface documentation and scoliosis screening. First evaluation results are highly promising.

## References

[B1] Boice JD Jr. Carcinogenesis--a synopsis of human experience with external exposure in medicine Health Phys 1988 55 4 621 630 10.1097/00004032-198810000-00003 3049457

[B2] Nash CL Jr. Gregg EC Brown RH Pillai K Risks of exposure to X-rays in patients undergoing long-term treatment for scoliosis J Bone Joint Surg Am 1979 61 3 371 374 429405

